# Pseudorenal Failure Secondary to Reversed Intraperitoneal Autodialysis

**DOI:** 10.1155/2013/982391

**Published:** 2013-12-25

**Authors:** Pieter Martens

**Affiliations:** Department of Internal Medicine, University Hospital Leuven, Herestraat 49, 3000 Leuven, Belgium

## Abstract

A 16-year-old boy was admitted for anuria, ascites, and abdominal pain. The patient had undergone a laparoscopic appendectomy two days prior to admission. Initial laboratory analysis revealed a plasma creatinine level of 5,07 mg/dL and blood urea nitrogen level of 75 mg/dL. Computed tomography imaging revealed diffuse abdominal ascites with normal kidneys without signs of hydronephrosis. Laprascopic revision found a 3 mm bladder tear and yielded an aspirate of 1,8 litre abdominal fluid. The abdominal fluid exhibited a fluid : serum creatinine ratio exceeding 1, indicating uroperitoneum. This case underscores the importance of bladder ruptures causing uroperitoneum presenting with azotemia.

## 1. Introduction

Bladder rupture with the development of uroperitoneum is a rare cause of ascites, abdominal pain, and azotemia. Here, we present a case of a young man with an iatrogenic bladder rupture following laparoscopic surgery.

## 2. Case Report

**Figure 1:**
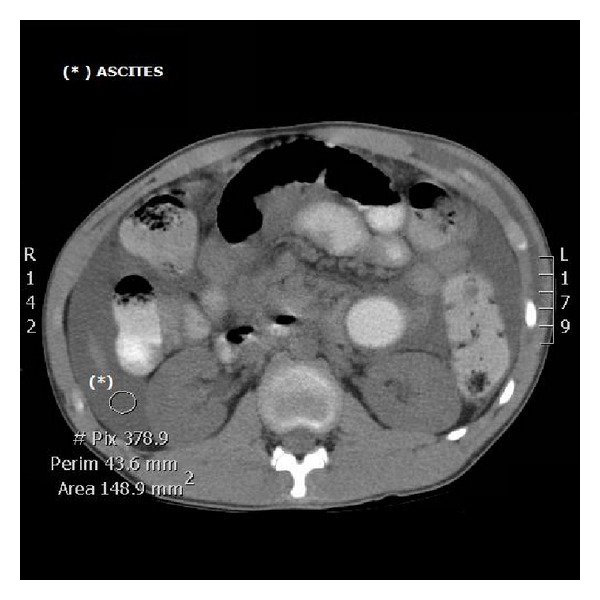
Axial CT slice demonstrating free fluid in the peritoneal cavity. The slide was made at the level of the kidneys that do not show hydronephrosis.

**Figure 2:**
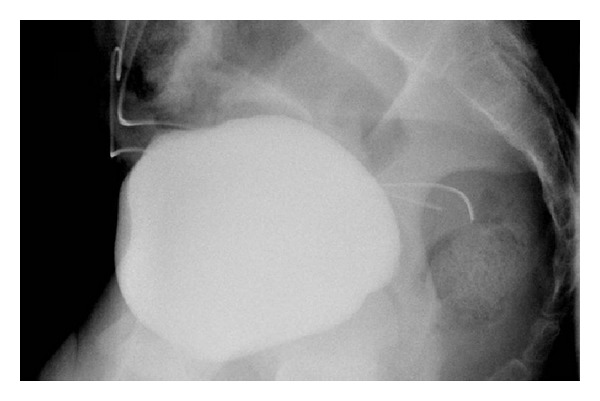
Retrograde cystography demonstrating normal integrity of the urinary bladder (no contrast leakage).

A 16-year-old presented to the emergency department with a complaint of inability to micturate, progressive abdominal dissention, and lower abdominal pain. The patient had undergone a laparoscopic appendectomy 2 days earlier and was discharged after a brief stay. Since his discharge, he was unable to micturate. His medical and surgical history is otherwise uneventful. He denies any recent trauma, alcohol use, or illicit drug use. He had been taking paracetamol and ibuprofen for postoperative discomfort. Physical examination revealed a young male in no acute distress. There was a distended abdomen, with shifting dullness. The lower abdomen quadrants were sensitive to palpation with rebound tenderness. There was no sign of globes vesicles. There was no asterixis or signs of hepatic disease. Urogenital and cardiopulmonal examination were unremarkable. Extremities were without edema. Vital signs were all within normal range (blood pressure: 115/80 mm Hg, no orthostatic hypotension, hearth rate: 85/min, temperature: 37.1°C, and respiration rate: 11/min). Laboratory analysis disclosed a plasma creatinine level of 5.07 mg/dL and a blood urea nitrogen level of 75 mg/dL, whilst creatinine and blood urea nitrogen concentration at discharge two days earlier were within normal range (creatinine level of 0.88 mg/dL and blood urea nitrogen level of 32 mg/dL.). Other laboratory results were within normal range. Abdominal ultrasound revealed diffuse abdominal ascites. There were no signs of hydronephrosis, and bladder volume was calculated to 170 mL. A CT-scan confirmed a significant amount of abdominal ascites. Intravesicular content exhibited approximately the same Hounsfield units as intraperitoneal content. There were no signs of hydronephrosis, and the kidney appeared normal (Figure 1). We postulated that the intraperitoneal fluid was urine secondary to a per-operative complication during laparoscopic appendectomy two days earlier. Although the exact nature of the kidney failure remained obscure. a laparoscopic reevaluation was planned the same day. Insertion of the laparoscopic trocar yielded clear no hemorrhagic fluid. A total of 1,8 litre of abdominal fluid was aspirated. Subsequent exploration revealed a 3 mm bladder tear, which was sutured laparoscopically. A Foley catheter was placed and left in situ for the ensuing five days. Postoperative analysis of peritoneal fluid revealed a fluid creatinine of 9 mg/dL and urea of 90 mg/dL. Peritoneal fluid : serum creatinine ratio was calculated to be >1.0, confirming the diagnosis of intraperitoneal urine leak. A follow-up blood analysis 12 hours after revision showed a normalised plasma creatinine level of 1,00 mg/dL. A follow-up retrograde cystography, five days after revision, was unable to demonstrate any remaining urine leakage (Figure 2). The patient was discharged in a well condition.

## 3. Discussion

The manifestation of bladder lesions differ according to the location of the tear in the bladder wall. Because the bladder has both an intraperitoneal and an extraperitoneal component, urine can extravasate in both compartments, generating different symptoms. Extraperitoneal perforation is most often associated with blunt trauma, presenting as suprapubic pain, edema, and oliguria [1–3]. This case reports a young man with an iatrogenic tear in the intraperitoneal component of the bladder, generating progressive urinary ascites. Intraperitoneal leakage of urine typically manifests abdominal pain, progressive urinary ascites, and oliguria/anuria [4, 5]. Iatrogenic puncturing of the bladder during laparoscopy is a known, but rare, complication and happens most often during insertion of the trocar or veress needle [6]. Retrospective analysis of large cohorts indicates an incidence of bladder injury during laparoscopy varying between 0,014% [7] 0,037% [6]. Tears in the bladder are most often found per-operatively, but they could be missed as illustrated by this case. Still there was a clear temporal relationship between the onset of symptoms and the surgery. This is not always true, as illustrated by reports of spontaneous bladder rupture. Spontaneous ruptures of the bladder present often later than the predisposing event. They are characterized by disease entities which all generate a pathological bladder wall weakness with or without an increased bladder pressure [1]. The dome of the bladder, which is only covered by peritoneum, is the predilection site for spontaneous bladder rupture. Conditions associated with spontaneous bladder rupture are radiotherapy for pelvic malignancy [5, 8], bladder cancer [2], bladder surgery [2], a neurogenic bladder [1, 9], bladder infection [10], alcohol [4, 11], and other drugs such as sympathomimetic agents [12].

But irrespective of the exact cause of intraperitoneal urine leakage (iatrogenic or spontaneous bladder rupture), progressive urinary ascites with the development of abdominal discomfort will soon follow. This is because the excretion function of the kidney greatly exceeds the subdiaphragmatic lymph flow, which provides the principal means for the removal of intraperitoneal fluid [11]. Stasis of urine in the peritoneal cavity allows for reversed intraperitoneal autodialysis to take place. The higher concentration of creatinine and nitrogenous waste products in the urine as compared to plasma allows for concentration gradient diffusion when the urine is in contact with the peritoneum, functioning as a semipermeable membrane [1, 3, 4]. As illustrated in this case, the patient's serum creatinine will rise. But because the glomerular filtration rate is intact, the raise of serum creatinine is referred to as pseudorenal failure [11]. Most physicians are unfamiliar with the entity of pseudorenal failure. Pseudorenal failure should be added to the differential diagnosis of patients with progressive abdominal ascites, abdominal pain, and a laboratory result indicative of acute azotemia. When pseudorenal failure based on reversed autodialysis is suspected, a peritoneal fluid analysis with measurement of creatinine can indicate the presence of urine in the abdomen [13]. This is when the peritoneal fluid : serum creatinine ratio exceeds 1, as was illustrated by this case. A cystography identifies a bladder tear by documenting intraperitoneal contrast leakage. Because cystography is not readily available in the emergency department, we performed a CT-scan which confirmed the presence of abundant intraperitoneal fluid, with grossly the same Hounsfield units as the intravesicular content. Laparoscopic revision with the suturing of the bladder defect and placement of a Foley catheter is proposed by the literature as a successful mean of treatment. This case confirms the efficacy of such treatment as illustrated by the pristine follow-up cystography. The case underscores the importance of the entity of pseudorenal failure. Removal of the intraperitoneal urine allows for a quick and spontaneous resolution of the laboratory results.
